# Formal Comparison of Dual-Parameter Temporal Discounting Models in Controls and Pathological Gamblers

**DOI:** 10.1371/journal.pone.0047225

**Published:** 2012-11-30

**Authors:** Jan Peters, Stephan Franz Miedl, Christian Büchel

**Affiliations:** NeuroimageNord, Department of Systems Neuroscience, University Medical-Center Hamburg-Eppendorf, Hamburg, Germany; University of Bologna, Italy

## Abstract

*Temporal* or *delay* discounting refers to the phenomenon that the value of a reward is discounted as a function of time to delivery. A range of models have been proposed that approximate the shape of the discount curve describing the relationship between subjective value and time. Recent evidence suggests that more than one free parameter may be required to accurately model human temporal discounting data. Nonetheless, many temporal discounting studies in psychiatry, psychology and neuroeconomics still apply single-parameter models, despite their oftentimes poor fit to single-subject data. Previous comparisons of temporal discounting models have either not taken model complexity into account, or have overlooked particular models. Here we apply model comparison techniques in a large sample of temporal discounting datasets using several discounting models employed in the past. Among the models examined, an exponential-power model from behavioural economics (CS model, Ebert & Prelec 2007) provided the best fit to human laboratory discounting data. Inter-parameter correlations for the winning model were moderate, whereas they were substantial for other dual-parameter models examined. Analyses of previous group and context effects on temporal discounting with the winning model provided additional theoretical insights. The CS model may be a useful tool in future psychiatry, psychology and neuroscience work on inter-temporal choice.

## Introduction

*Delay* or *temporal* discounting refers to the phenomenon that humans and many animals discount reward value over time [1]. A reward of 100€ available in two weeks is subjectively of lesser value than the same amount available immediately. The subjective value of a reward therefore decreases with increasing delay. Decisions between outcomes that are separated in time (“inter-temporal choice”) are abundant in everyday life, and across a range of disciplines, there is continually growing interest in such decision processes, e.g. in economics [2], psychology [1], psychiatry [3], [4] and cognitive neuroscience [5], [6]. A range of models have been proposed that approximate the shape of the discount function relating reward value to delay [7]–[15] or reward value to the inter-reward-interval [16]–[18], the most frequently used being single parameter exponential and hyperbolic models.

In standard hyperbolic discounting [9] (Table 1 Eq. 1), the degree of discounting is a function of the delay, such that discounting is relatively steeper over time intervals in the near future than over time periods in the far future, giving rise to the phenomenon of “decreasing impatience” [12]. In contrast, in the exponential “discounted utility” model from classical economics [8] it is assumed that decision-makers behave rationally in the sense that the effect of a particular delay is independent of the point in time when that delay occurs. According to the exponential model (Table 1, Eq. 2), a waiting period of 1 week from today should be treated the same way as a waiting period of 1 week in a year's time. In contrast to this prediction of “normative” exponential discounting, and in line with hyperbolic discounting, humans often violate this assumption, an effect that has been termed “dynamic inconsistency” [19], the “common difference effect” [11], or “non-stationarity”. In line with this effect, it is well replicated that the hyperbolic model fits temporal discounting data better than the exponential model [1], [10], [14], [16], [20]–[23].

**Table 1:** Model equations for five prominent models of inter-temporal choice; *SV* – subjective (discounted) value, *A* – reward amount, *D* – delay.

Model	Abbreviation used in text	Equation
Hyperbolic	H	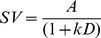
Exponential	E	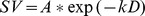
Green & Myerson (1995)	GM	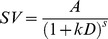
Mazur (1987), Rachlin (2006)	R	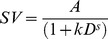
Ebert & Prelec (2007)	CS	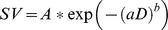

Recent studies suggest that more than one free parameter is likely required to accurately model human temporal discounting data [10], [24]. In particular, both the hyperbolic and the exponential models typically over-estimate discounted values at shorter delays and under-estimate discounted values at longer delays [10]. The oftentimes poor fit of these standard models to single-subject data has lead researchers to propose criteria for identifying (and excluding) problematic datasets [25] or endorse the use of more flexible models [13]. A practical side of this issue is that temporal discounting is increasingly examined in a neuroeconomic context, where predictors derived from discounting models are used in the analysis of neural data, e.g. neuroimaging data or electrophysiological recordings [21], [22], [26], [27]. In such studies, the number of subjects is typically limited, exclusion is costly, and adequate modelling of single-subject data is therefore essential (see also Bleichrodt et al., 2009).

At the same time, more adequate modelling of behaviour may yield additional theoretical insights into the mechanisms underlying the decision process. A classical view considers delay discounting as a self-control problem [28] which depends on (lateral) prefrontal cortex control regions [5], [29]–[31]. However, additional processes likely contribute to the phenomenon [32]. For example, how objective time is processed may affect the degree of discounting [33]–[38]. That is, non-linear scaling of objective time may, like hyperbolic discounting, give rise to “decreasing impatience” [35]–[37], resulting in a greater relative impact of proximal compared to more distal temporal intervals. A number of multi-parameter discounting models address this issue by modelling subjective time as a power-function of objective time [7], [14], [24], [36], [39], [40]. Along similar lines, non-linear scaling of reward magnitude has been incorporated in some temporal discounting models [7], [41]. However, these models are beyond the scope of the present paper. A desirable property of a temporal discounting model with such a psychophysical temporal scaling component would therefore be the potential possibility to disentangle effects of impatience (i.e. the steepness of the discounting function) from effects of temporal psychophysics (i.e. whether subjective time is expanded or contracted). However, this would require that the respective model parameters are largely independent, an issue that previous model comparison efforts have not addressed [10], [24], but see [12].

Equations 3 and 4 from Table 1 show two modifications of the single-parameter hyperbolic model, both of which include an additional scaling exponent *s* at different positions in the denominator. Equation 3 [20] raises the entire denominator of the standard hyperbolic model to a power *s*. As shown by Takahashi and colleagues [24], this model is equivalent to exponential discounting with logarithmic (i.e. Weber-Fechner) scaling of objective time. This model is also a special case of the generalized hyperbola proposed by Loewenstein & Prelec [11]. Equation 4 [9], [15] raises only the delay to a power *s* and thus corresponds to hyperbolic discounting with power-scaling of objective time [39], [42]. Note that this function is a special case of the initial formulation of a discounting-by-intervals function [40], in which the delay to the smaller-sooner reward is 0.

Ebert & Prelec (2007) proposed a “constant sensitivity” (CS) discounting function (Equation 5) based on a consideration of decision heuristics. In this model, the *a*-parameter measures the level of impatience, while time sensitivity is measured by the b-parameter. Exponential discounting corresponds to the special case *b* = 1. This model can account for a “present-future dichotomy” heuristic [12], where all future rewards are similarity down-weighted relative to the immediate present, by a small *b* (*b*<<1). In contrast, an “extended present” heuristic in which all options up to a particular delay are not discounted, and all later options are discounted to a similar degree can be captured by a large *b* (i.e. *b*>>1). Note that (as discussed above) *b* could also be interpreted as governing the power-scaling of time [see also Killeen (2009)], similar to other models with a scaling exponent [1], [14], [39]. In this sense, what Ebert & Prelec call “present-future dichotomy” (*b*<<1) may arise because the relative impact of short vs. long delays is enhanced due to a *compressed* time scale. The “extended present” (*b*>>1), on the other hand, may arise because the relative impact of long vs. short delays is enhanced due to an *expanded* time scale. Note that this model is a special case of a family of discount functions later described by Bleichrodt et al. [13].

Finally, the so-called “beta-delta” model accounts for dynamic inconsistency through an “immediacy effect” such that all outcomes that are not available *now* are discounted according to discount rate β, whereas all further (non-immediate) discounting occurs according to the discount rate δ [43]. This model has received particular attention based on the idea that the two model components (β and δ) may have distinct neural substrates [3], [30], [44]. Nonetheless, such an “immediacy effect” may not be sufficient to account for human behavioural data [23]. Note also that the CS model encompasses the continuous-time version of the beta-delta model (Ebert & Prelec, 2007, Proof in Appendix), which is therefore not included as a separate model in this report.

We note that a number of previous comparisons of temporal discounting models have been conducted [10], [15], [20], [24]. With the exception of the Takahashi et al. study [24], these studies have focussed on variance-accounted-for (R^2^) as a measure of goodness-of-fit. This approach is problematic for at least two reasons. First, this analysis confounds goodness-of-fit with the discount rate, at least in the single-parameter case, because R^2^ and *k* are positively correlated [25]. This is because steeper discounting is associated with a greater deviation of the indifference points from unity. As a consequence, the fit of the non-linear regression relative to the mean of the data (R^2^) increases with increasing discount rate. Second, solely relying on goodness-of-fit for model comparison is problematic when models have different numbers of free parameters, as model complexity is not accounted for in the model selection procedure, giving rise to the over-fitting problem [45]. To avoid over-fitting, measures such as the Akaike Information Criterion (AIC) [46] or the Bayesian Information Criterion (BIC) [47] can be used, which include a penalty term for increasing model complexity, thereby balancing parsimony and goodness-of-fit [45], [48]. Nevertheless, the only study that used such an index (AIC) to compare temporal discounting models [24] did not examine models 4 and 5 (see Table 1), making a direct comparison to the data of McKerchar et al. (2009) difficult.

Data from temporal discounting paradigms are typically available in two types of formats. Many classical studies are based on a relatively small set of around 6–8 indifference points (i.e. points of subjective equivalence in value of smaller-sooner [SS] and a larger-later [LL] reward, for a particular delay), and curves are fit to these data points using non-linear regression [10]. In contrast, many neuro-economic investigations of temporal discouting give rise to trial-by-trial discounting data, i.e. relatively large sets of choices between SS and LL rewards across different combinations of delays and amounts [16], [22], [26], [49]–[51]. These datasets can also be analyzed using curve fitting, although some method needs to be implemented to estimate the indifference points from trial-by-trial data [22], [26]. Here we use an alternative approach based on maximum likelihood estimation (MLE) and fit the model to data from each participant in all trials [52].

Steep temporal discounting is a hallmark of many psychiatric conditions, including substance abuse and addiction [3], [5]. However, surprisingly little is known about differences in the shape of the discount function between addicts and controls. Earlier studies revealed a superior fit of the hyperbolic vs. exponential model in both healthy controls and addicts [53], [54], and the overall fit of the hyperbolic model appears to be similar in addicts and controls [50], [55]. Nonetheless, dual-parameter discounting models have to our knowledge not been examined in addicts [though see Killeen (2009) for a re-analysis of previously published group-aggregate data].

Aims of this study were therefore first to compare goodness-of-fit for a number of prominent temporal discounting models while correcting for differences in complexity. Second, we examined whether the same model accounts for data from controls and pathological gamblers, a clinical group known for high trait impulsivity. We fit several prominent models of delay discounting (Table 1) to a large number of datasets from healthy controls (n = 198) and pathological gamblers (n = 17). We applied Maximum Likelihood parameter estimation and used the AIC as a measure of goodness-of-fit [46]. Analyses are complemented with a Bayesian Model Selection procedure [56] that treats the underlying model as a random effect across subjects, and is therefore less prone to outliers than model selection solely based on AIC.

## Methods

### Ethics statement

For all data re-analyzed in the present paper, informed written consent was acquired from participants prior to participation. All study procedures were approved by the local ethics committee (Institutional Review Board of the Hamburg Physicians Association).

### Included data

We re-analyzed delay discounting data from three previously published behavioural and functional magnetic resonance imaging studies of delay discounting [26], [49], [50] as well as previously unpublished behavioural data. Two types of datasets are included in the analysis: Firstly, we re-analyzed behavioural data from three functional magnetic resonance imaging studies (Exp. 1 from Peters & Büchel, 2009, n = 22, Exp. 1 from Peters & Büchel, 2010, n = 30, data from Miedl et al., 2012: n = 18 control subjects [note that two of these subjects were not included in the original paper because their matched pathological gamblers had to be excluded]) and one behavioural study of delay discounting (n = 16, Exp. 2 from Peters & Büchel, 2010) were analyzed (referred to henceforth as “dataset 1”). Data from n = 17 pathological gamblers (referred to henceforth as “pathological gamblers”) from a previous study are included [50] to compare the findings in healthy participants to a clinical group that is well known for their impulsive discounting behaviour [50], . In dataset 1 and pathological gamblers, subjects made repeated choices between immediate rewards of 20€ and larger but delayed amounts. Choices involved the possibility for real gains, as one trial was randomly selected following testing, and paid out for real, either using gift certificate from an online shop [26] or using timed bank transfers [49], [50]. In addition, all presented offers were pre-determined based on subjects' performance on a previous adaptive delay discounting task (as in dataset 2, see next paragraph). The procedure for calculating individual offers is described in detail elsewhere [26], [49], [50]. In short, we estimated the standard hyperbolic discount rate for an adaptive delay discounting task for each participant. Based on this estimated discount rate, we calculated indifference amounts for each delay (i.e. amounts that were subjectively equivalent in value to the immediate reward of 20€). The minimum amount was set to 20.5€ and the maximum amount to 80€. For each delay, equal numbers of uniformly distributed offers were created with an estimated subjective value lower (50% of offers) and higher (50% of offers) than the indifference amount. Delays for the Peters & Büchel (2009) datasets were 0.25 days, 1 day (d), 7 d, 30d, 90d, 180d. Delays for the Peters & Büchel (2010) datasets were similarly spaced but included one additional delay. Also, they differed slightly between subjects (see Peters & Büchel, 2010 for details), because they were selected individually to be non-overlapping with delays to subject-specific future events, which were used in a separate experimental condition [49]. Importantly, for consistency with dataset 2, for the Peters & Büchel (2010) subjects we only include data from the standard delay discounting condition without future event cues in the model comparison.

Secondly, data from an adaptive delay discounting paradigm were analyzed (n = 112 datasets, referred to henceforth as “dataset 2”). This task was administered to all subjects at multiple time points to assess the stability of temporal discounting over time. We only include data from each participant's first testing session. In this task, subjects made repeated choices between 20€ available immediately and larger but delayed hypothetical amounts of money (delays [days]: 1, 2, 7, 14, 30, 90, 180). The hypothetical rewards always amounted to at least 20.5€, but without an upper limit. An adjusting-amount procedure was used such that, following two successive choices of the delayed reward, the delayed amount was reduced, and following two successive choices of the immediate reward, the delayed amount was increased in a step-wise manner. The algorithm terminated as soon as the difference between accepted and rejected delayed amounts reached a delay-specific criterion [Criterion in €: 1.0 (1d), 1.5 (2d), 2.0 (7d), 2.0 (14d), 3.0 (30d), 4.0 (90d), 4.0 (180d)] [26].

### Model fitting and comparison

#### Non-linear least squares and R^2^

Using R^2^ for model selection is problematic in particular because of the problem of over-fitting [45], and because R^2^ confounds goodness-of-fit with the discount rate [25]. For comparison with previous studies [10], [20] we nonetheless include results from a “classical” analysis of the fit of discounting models to indifference point data. We examined median indifference points from two subsets of dataset 2 (see above, i.e. subjects with 6 and subjects with 7 indifference points). Model equations (see Table 1) were fit to these group-aggregate indifference points using non-linear least squares as implemented in Matlab © (*lsqcurvefit*) and we report R^2^ as a measure of goodness-of-fit. We used the same method to model single-subject indifference point data and report the distributions of these values. R^2^ values between dual-parameter models were compared using Wilcoxon signed rank tests.

#### Maximum-likelihood estimation (MLE)

**Figure 1:**
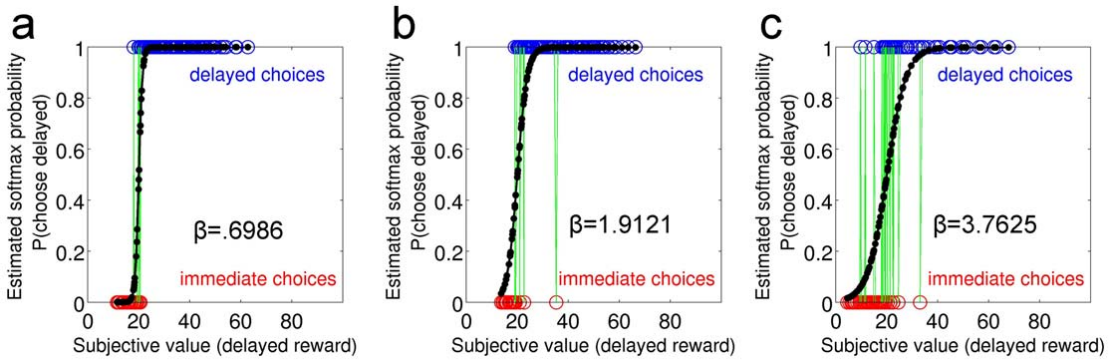
Actual choices of three exemplary subjects from dataset 1 (a–c). Red circles indicate choices of the immediate reward, whereas blue circles indicate choices of the delayed reward. The x-axis shows the subjective discounted value of the delayed reward, in this particular case calculated using Eq. 4 (R model). The y-axis plots the estimated probability of choosing the delayed reward based on the softmax choice function [P(choose delayed), black solid line] calculated using the best-fitting model parameters for that particular subject. Each green line indicates an “inconsistent” choice that is not predicted by the model. The steepness of the sigmoid softmax function (plotted in black) is modelled by the temp-parameter. Subjects with low (a, β = 0.6986), moderate (b, β = 1.9212) and high (c, β = 3.7625) degrees of stochastic responding are displayed to illustrate inter-subject variability. Note how increasing numbers of inconsistent choices are reflected in an attenuated steepness of the sigmoid.

For the remaining model comparison procedures, we applied maximum-likelihood estimation [48] with optimization procedures implemented in Matlab © (*fminsearch*) to obtain the best-fitting parameter estimates for each individual participant. Note that this procedure uses trial-by-trial data [52]. Specifically, we applied the softmax choice rule
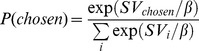
(6)to estimate the probability of choosing the selected option (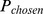) on each trial, given the subjective values of the available options *i*. These subjective values (SV) correspond to values of the smaller/sooner reward and larger/later rewards according to the particular discounting model under consideration. Because the immediate reward was fixed in all experiments, the value of this option was a constant (20€). Here, the free parameter *β* models the stochasticity of a participants' choice behaviour, given a particular discounting model, i.e. the steepness of the sigmoid choice function. This choice function is frequently applied in computational modelling of reinforcement learning [60], [61] and decision-making, including delay discounting [34], [41], [50], [51], [62], [63] to convert value differences between decision options into choice probabilities. To illustrate the use of the softmax function, Figure 1 shows data from three individual subjects with different degrees of stochastic responding (i.e. different slopes [*β*-parameters] of the sigmoid). Note that for Figure 1, the subjective values plotted on the x-axis were calculated using Equation 4, the R model. Note as well how increasing numbers of choices that are inconsistent with a participant's ML parameter estimates result in a decrease in the steepness of the sigmoid and thus in an increase in *β*.

To obtain the best-fitting model parameters for each model and subject, we then maximized the log-likelihood (LL) of the choice probabilities (i.e. 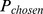 from Eq. 6) given a particular set of model parameters θ, summing across all trials *t* for each subject:
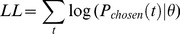
(7)


To avoid local minima, the estimation procedure was repeated with 20 random combinations of starting values for each model in each subject, while keeping track of the overall maximum *LL*. As measure of goodness-of-fit we calculated the Akaike Information Criterion (AIC) [24], [46]:
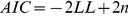
(8)


Here, *n* is the number of free parameters in the model and LL is the log-likelihood from equation (7). Because the relative differences in model fit, rather than the absolute AIC scores, are of interest [64], we calculated ΔAIC values for each model *M* as



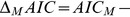
(9)


That is, the AIC of the best-fitting model is subtracted from each models' AIC value. This is done at the group level (for the summed AIC values across subjects) and for each individual subject, yielding a ΔAIC of 0 for the best-fitting model. Statistical comparisons were conducted on the single-subject ΔAIC values using non-parametric Wilcoxon Signed Rank tests to account for the skewed distribution of these measures. We also performed all analyses using the Bayesian Information Criterion BIC instead of AIC [47]. However, since results were largely comparable to the analyses using AIC, these data are not reported here.

#### Bayesian model selection

Finally, we applied Bayesian Model Selection (BMS) [56], [65] as implemented in the software package Statistical Parametric Mapping (SPM) version 08-4267 (Wellcome Department of Cognitive Neurology, University College London). In short, this model comparison procedure treats the underlying model *M* as a random variable across subjects and assumes that different subjects may have different generating models. In contrast to a consideration of ΔAIC scores (see previous paragraph), in the BMS approach the influence of outliers has a natural bound, because the belief that model *M* generated the data of a particular subject can not exceed unity [56]. Notably, this procedure requires only the log-model-evidences of each model as input, which can be approximated with the AIC or BIC [56]. The BMS analysis computes an *exceedance probability* for each model, which is the probability that a particular model is more likely than any other of the models tested to have generated the data of a randomly selected subject from the population. BMS has been previously applied to model selection in the context of reward-based decision-making [65]. In line with this previous study, we consider an exceedance probability of P>95% to be decisive.

#### Inter-parameter correlations

We examined dependencies between model parameters using the following procedure. For each subject, the Hessian matrix (i.e. the matrix of second derivatives of the likelihood function) was estimated using numerical finite difference approximation [48],[66],[67] with the step-size set to 10^−3^. The inverse of the Hessian was taken to obtain the covariance matrix [48], and subsequently converted to a correlation matrix using the Matlab function *corrcov*. Correlations between parameters were then averaged across subjects.

Note that in some cases the obtained Hessians may be non-invertible, precluding one from obtaining a covariance matrix. This can occur if the ML estimates lie close to a parameter boundary (e.g. zero) or if they are located on a plateau or ridge of the surface of the likelihood function [68]. Because the *a*-parameter in the CS model often takes on values close to 0, the problem was particularly pronounced for this model (non-invertible Hessians in dataset 1: 29/86, dataset 2: 28/112, gamblers: 2/17). We therefore re-fit the CS model using a re-scaling of the *a*-parameter (i.e. we fitted *a*/100 rather than *a*) which effectively reduced the number of subjects with non-invertible Hessians (dataset 1: 11/86, dataset 2: 12/112, gamblers: 1/17).

#### Group differences and context effects

We examined previously described group and context effects in temporal discounting using the winning model. To this end, we compared model parameters from the gamblers to a set of n = 18 matched controls [50]. We also examined our previously published data on effects of episodic future thinking on temporal discounting. Here we compared model parameters from a temporal discounting condition in which subject-specific episodic event cues were presented in addition to delays, to a control condition without such event cues [49]. Note that the skewed distribution of discounting model parameters typically requires transformation before parametric statistics can be applied [25], [49], [50]. Here we used a square-root transformation [69] that has the advantage that the transformed values have a lower bound of 0. In contrast, the commonly applied log-transformation can cause problems with parameter values that approach zero, because 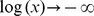
as 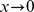.

## Results

### General approach

Medians and inter-quartile ranges of single-subject maximum likelihood parameter estimates are listed in Table 2 for each model. We applied several model comparison procedures: 1) comparison of R^2^ based on model fits to group-aggregate and single-subject indifference points [10], 2) examination of group aggregate [41] and mean individual ΔAIC scores 3) a random-effects Bayesian model comparison [56], [65]. The first approach was included primarily to improve accessibility of this report for researchers more familiar with more classical model selection procedures, and because this procedure is still widely applied in the psychological literature on temporal discounting [10], [15], [20]. The second approach alleviates the main problem associated with model comparison based on R^2^, over-fitting [45], because the AIC includes a penalty term for model complexity. Nevertheless, both approaches can be influenced by outliers, and both essentially attempt to determine the model that best fits the data of all subjects. In contrast, Bayesian model selection (BMS) is a recently developed approach [56] that computes an exceedance probability for each model, i.e. the probability that a given model is more likely than any other model from the set to have generated the data of a randomly selected subject from the population. This approach therefore acknowledges that different subjects may have different data-generating models, but attempts to identify the most likely model in the population.

**Table 2:** Medians (M) and inter-quartile ranges (IQR) of maximum likelihood parameter estimates for the five discounting models examined (see Table 1 for model equations, numbers and abbreviations).

	Model parameters								
	β			*k/a*			*s/b*		
Dataset Model	1	2	PG	1	2	PG	1	2	PG
(1) H: Median (M))	2.16	4.29	2.66	.0083	.0112	.0463			
Inter-quartile range (IQR)	1.06–2.88	2.59–6.66	1.51–4.41	.0042–.0166	.0047–.0249	.0125–.144			
(2) E: M	2.44	6.59	3.32	.0055	.0055	.0314			
IQR	1.16–3.87	3.04–10.99	1.66–5.18	.0032–.009	.0029–.0097	.0073–.102			
(3)GM: M	1.36	2.56	1.82	.0434	.0991	.0778	.403	.543	.712
IQR	.72–1.93	1.46–3.51	1.09–2.45	.0064–.0409	.0087–.4231	.0047–.328	.174–.895	.159–1.03	.26–1.34
(4) R: M	1.36	2.28	1.77	.0366	.0573	.0749	.713	.751	.752
IQR	.65–1.97	1.38–3.51	1.02–2.43	.0068–.091	.01–.117	.0097–.191	.429–.938	.449–1.03	.467–.998
(5) CS: M	1.22	2.16	1.86	.0049	.0067	.0287	.562	.511	.681
IQR	.62–1.94	1.31–3.64	.86–2.69	.0015–.0084	.0028–.0162	.0068–.0592	.348–.755	.357–.779	.411–.899

Parameters are shown separately for the three different datasets (1, 2, pathological gamblers [PG]).

### Model comparison based on R^2^

**Figure 2:**
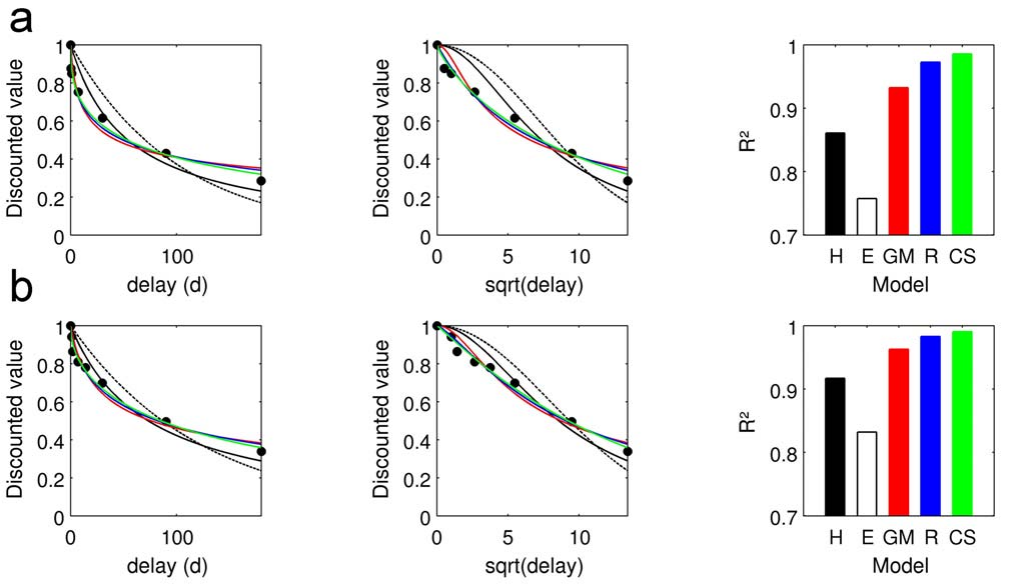
Discount function fit to median indifference points (a: n = 50, b: n = 55). Leftmost panels plot subjective discounted value against time, center panels plot value against time^1/2^ to improve visualization of the shorter delays. R^2^ for each model function is plotted in the rightmost panels.

Figure 2 plots the fits of the discounting models of interest to group-median indifference points, separately for datasets with six (top row, n = 50) and seven (bottom row, n = 55) indifference points. (Note that indifference points were unavailable for 7 additional subjects, which are nonetheless included in dataset 2 for the analysis using maximum likelihood estimation.) Value is plotted both against time (left) and time^1/2^ (center panel) to improve visualization of the shorter delays. It can be seen the dual-parameter models fit the data better than the single-parameter hyperbolic and exponential models [1], [10], [15]. As shown previously [10], it can be seen in the center panels of Figure 2 that the poorer fit of the single-parameter models was primarily due to an overestimation of value for shorter delays, and an underestimation of value for longer delays. Table 3 summarizes the results from the non-linear regression analysis. For the individual subject fits, the order of goodness-of-fit was CS>R>GM>H>E. Among the dual-parameter models, all paired comparisons of R^2^ values were significant (Wilcoxon signed rank tests, CS. vs. R: Z = −2.67, p = .0076; CS. vs. GM: Z = −4.57, p<.001; GM vs. R: Z = 5.29, p<.001).

**Table 3:** R^2^ values (medians and inter-quartile ranges) for the non-linear regression analysis of indifference point data for single-subject fits.

Model	R^2^ group fit (6-ID-points)	R^2^ group fit (7-ID-points)	Median (IQR) R^2^: individual subject data
H	.861	.917	.876 (.714–.943)
E	.758	.832	.814 (.607–.905)
GM	.933	.963	.953 (.912–.972)
R	.973	.982	.957 (.930–.979)
CS	.986	.988	.961 (.926–.983)

### Model comparison based on AIC

**Figure 3:**
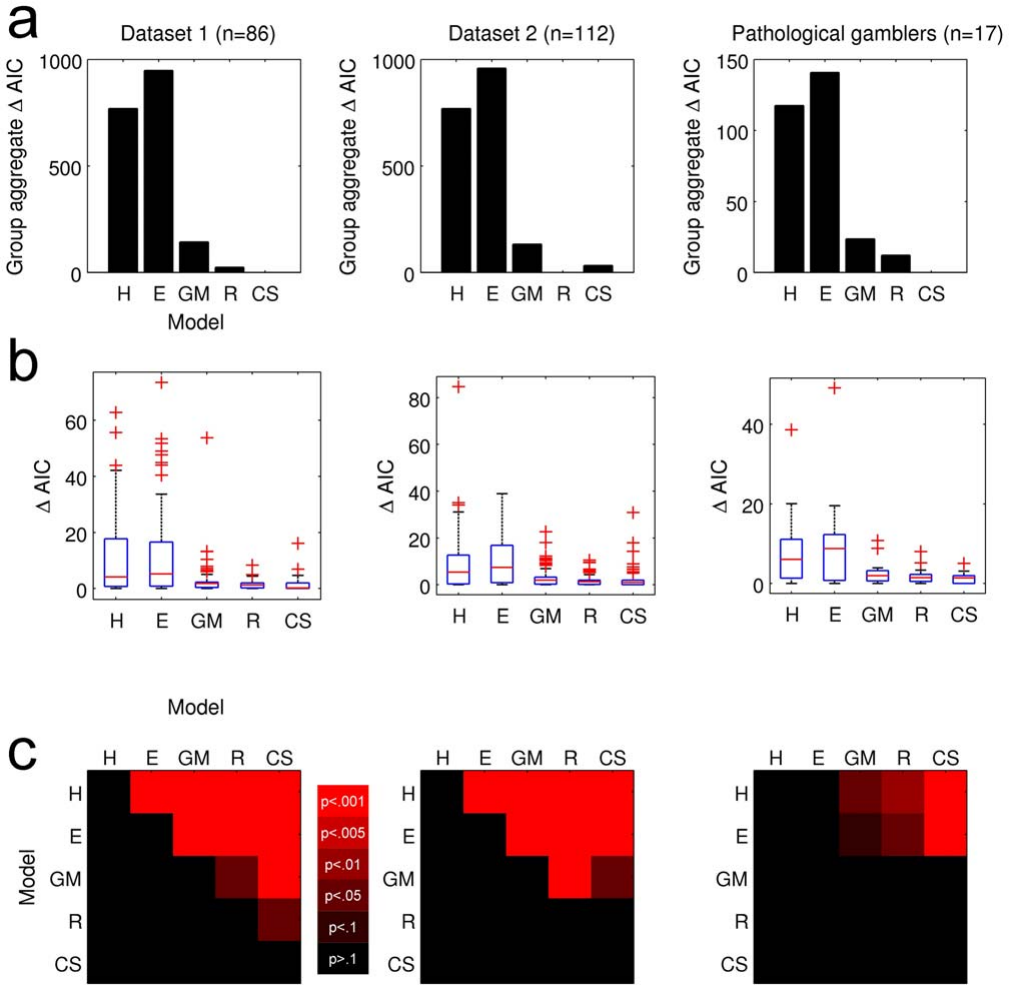
Model comparison based on AIC. a) Group-aggregate ΔAIC scores for each model were calculated by summing AIC scores over all subjects and then substracting each model's AIC score from the best-fitting model's AIC score, yielding a ΔAIC of 0 for the best-fitting model. b) Boxplots of single-subject ΔAIC values. c) P-values from Wilcoxon rank-sum tests conducted on ΔAIC values for each model pairing.

We next examined whether dual-parameter models improved the fit to discounting data over and above an increase in fit due to their additional complexity. To this end, we calculated ΔAIC scores (see methods section) for both group aggregate and single-subject data. All dual-parameter models had lower ΔAIC scores than the single-parameter models, both for group-aggregate data (Figure 3A) and single-subject data (Figure 3B). This confirms previous findings that greater R^2^ (see above) is not purely due to over-fitting [24], [45]. Amongst the dual-parameter models, the CS model showed the smallest group-level ΔAIC and smallest average single-subject ΔAIC scores for dataset 1 and pathological gamblers. For dataset 2, ΔAIC scores of the R model were slightly lower.

**Figure 4:**
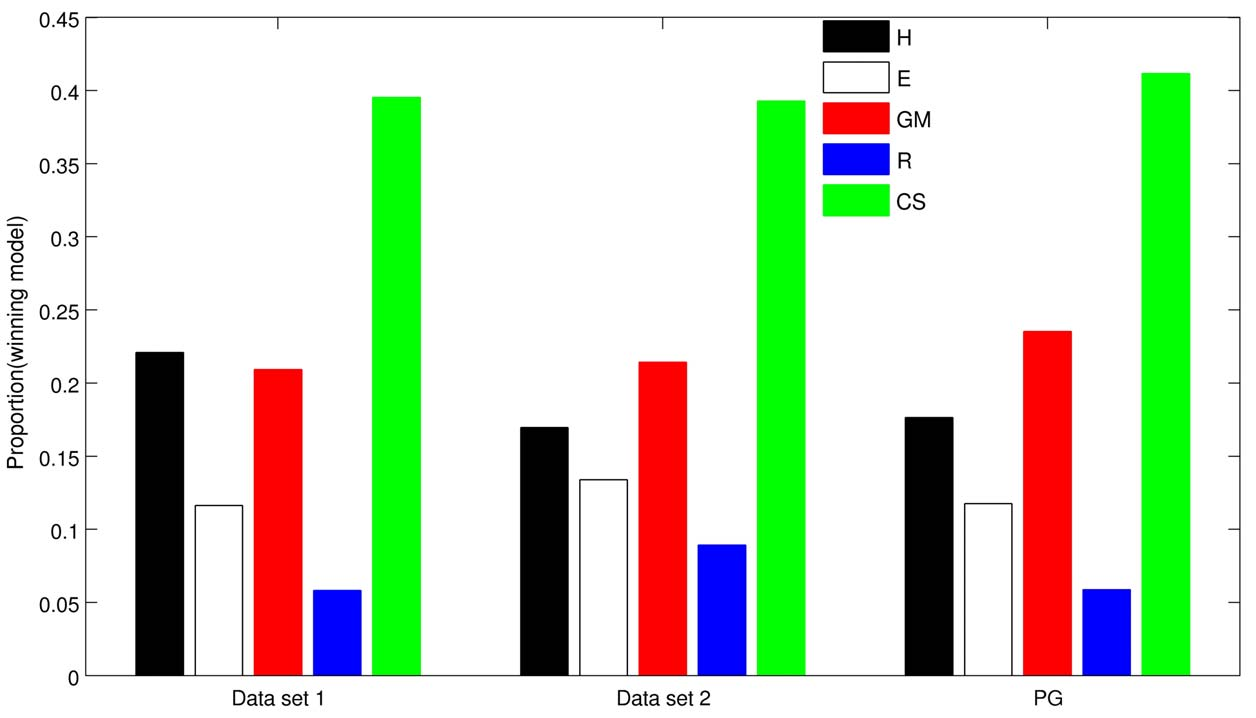
Proportion of subjects for whom each model provided the best fit, based on AIC. In all datasets, the CS model was the most frequent winning model, providing the best fit in roughly 40% of participants across datasets.

Figure 3C provides a statistical comparison of single-subject ΔAIC values for all model pairings and datasets using non-parametric Wilcoxon signed rank tests. Many of the direct comparisons even among dual-parameter models were significant. However, note that this analysis aims to identify the single best model across subjects. Yet, it can be seen from the box plots in Figure 3B that ΔAIC scores showed considerable variability between subjects (including outliers) which can influence model selection even if non-parametric statistics are applied [56]. Also, different subjects may have different data-generating models. We therefore next examined the proportion of participants for whom each model provided the best fit, based on the AIC. It can be seen from Figure 4 that the CS model was the most frequent winning model, providing the best fit in roughly 40% of participants across all datasets. To more formally quantify this observation, we conducted a Bayesian Model Selection procedure.

### Bayesian Model Comparison

**Figure 5:**
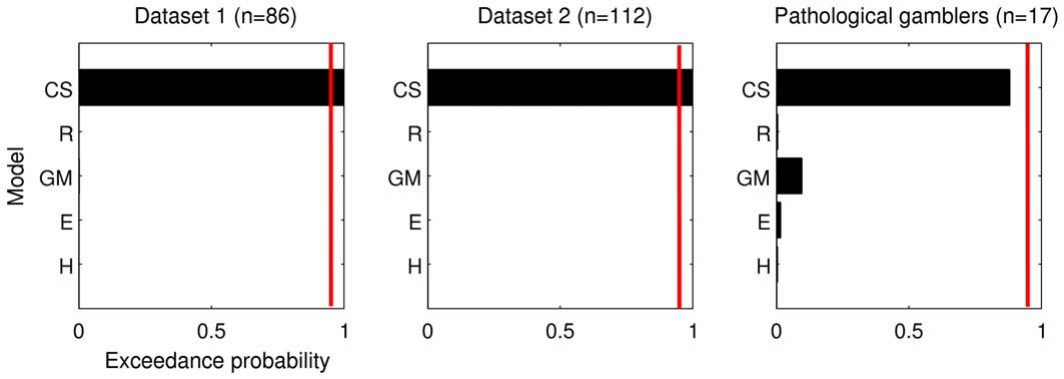
Results from the Bayesian model selection procedure [**56**] using the AIC as approximation to the log-model-evidence. Plotted are exceedance probabilities for each model (i.e. the probability that each given model is more frequent in the population than any other model in the set). The red line denotes p>95%.

AIC scores as an approximation to the log-model-evidence were submitted to a BMS analysis as implemented in the software package SPM-08. As can be seen in Figure 5, both for dataset 1 and dataset 2, the exceedance probability of the CS model was decisive (P>95%) suggesting that the CS is most likely to be most frequent in the population, out of the five models examined in this report. The CS model also had the highest exceedance probability in the dataset of pathological gamblers, although due to the small sample size it was not decisive (P≈90%). Note the difference in the conclusions that we can draw from the BMS analysis as opposed to the previous consideration of ΔAIC scores. The ΔAIC analysis suggests that the CS model accounts for the data best on average across subjects. In contrast, the BMS analysis, which places a natural bound on the influence of outliers (the confidence that model M generated the data for a particular subject cannot exceed unity) suggests that the probability that the CS model is more frequent in the population than any other model tested is decisive (i.e. p>95%).

### Scaling parameter ranges and inter-parameter correlations in dual-parameter models

As outlined in the introduction, exponents in temporal discounting models are often interpreted in terms of psychophysical scaling of time or reward magnitude [7], [10], [12], [15], [24], [39], which would entail exponent values <1 [39], [42]. In this sense, scaling parameters of 1 indicate a linear representation of the respective variable (e.g. linear subjective time). Smaller scaling exponents indicate non-linear scaling of the variable, such that the relative impact of smaller vs. larger values is increased. Scaling exponents above 1 indicate that the relative impact of larger vs. smaller values is increased.

We tested this hypothesis (exponent<1) statistically using Wilcoxon signed rank tests, and results are summarized in Table 4. In datasets 1 and 2, there was at least a trend for exponent values <1 for the R, GM and CS models. This effect appeared most pronounced for the CS model. We did not observe exponent values <1 in the pathological gamblers, likely due to smaller number of subjects.

**Table 4:** Results of non-parametric statistical comparisons (Wilcoxon signed rank test) of dual-parameter model exponent parameters to 1.

	Model		
	GM	R	CS
Dataset1			
Z-value	−1.76	**−4.60**	**−6.12**
P-value	.0786	**<.001**	**<.001**
Dataset2			
Z-value	**−2.33**	**−2.52**	**−3.73**
P-value	**.020**	**.0117**	**<.001**
Pathological Gamblers			
Z-value	−.261	−1.11	−1.21
P-value	.795	.266	.227

Please refer to Table 2 for the medians and inter-quartile ranges of these parameters.

**Figure 6:**
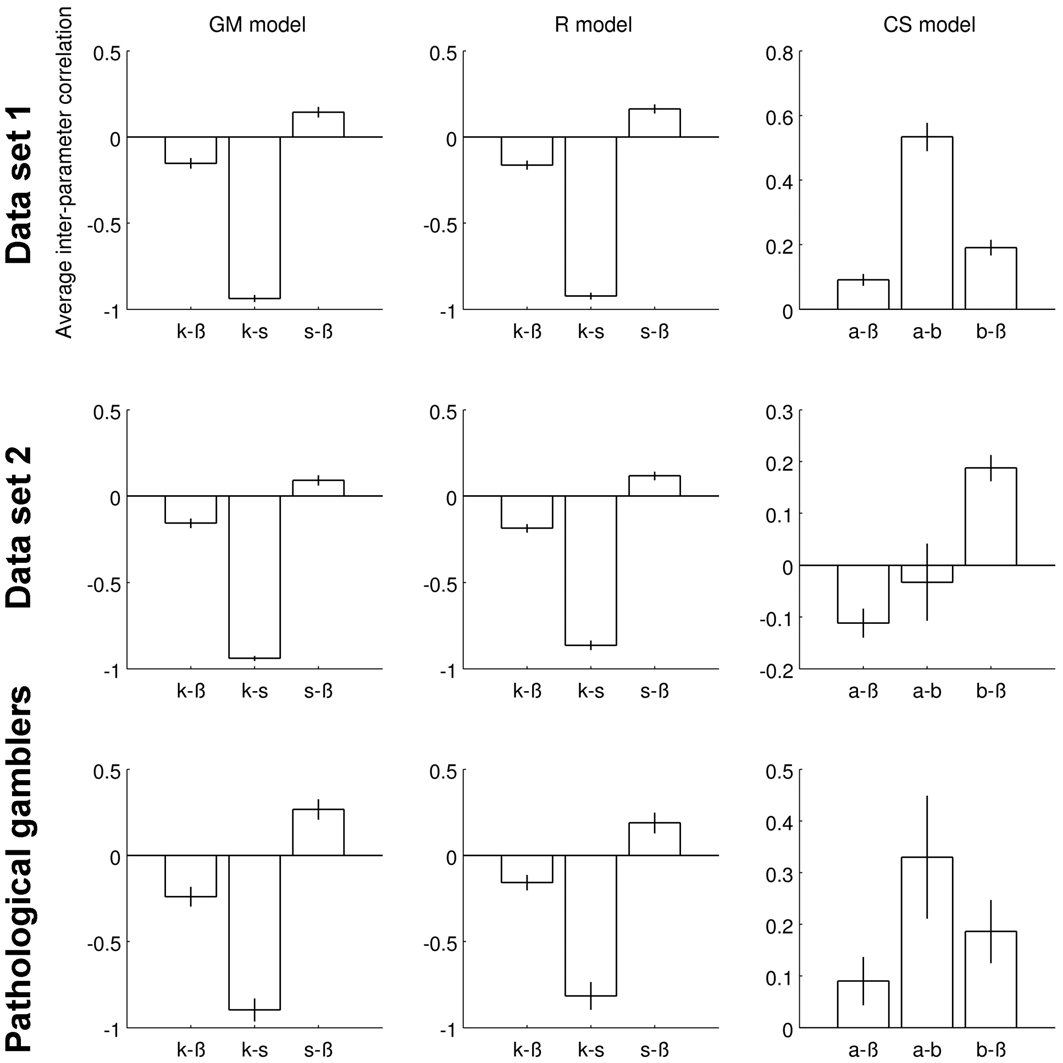
Within-subject inter-parameter correlations averaged across participants for each dataset. Note the strong inverse relationship between *k* and *s* in the R and GM models. In contrast, correlations between *a* and *b* in the CS model were much less pronounced. Correlations were computed based on a numerical approximation of the Hessian matrix (see methods section).

Although scaling exponents in temporal discounting models have been interpreted as reflecting distinct changes in underlying processes (e.g. time or magnitude scaling) based on fits to group-level data [see e.g. Killeen (2009)], how the different model-parameters are correlated has received relatively little attention [but see Ebert & Prelec (2007) for an analysis of between-subject inter-parameter correlations]. We therefore examined, for each dataset separately, the inter-parameter correlation for all dual-parameter models. This was done via numerical approximation of the Hessian matrix for each model and participant. The inverse of the Hessian is the covariance matrix, from which inter-parameter correlations can be calculated (see methods section for details). Figure 6 plots the average inter-parameter correlations for all dual-parameter models. It can be seen that *k* and *s* in the GM and R models showed a strong negative dependency. In contrast, the association between *a* and *b* in the CS model was considerably less pronounced.

### Sensitivity to group differences and contextual effects

Finally we examined the sensitivity of the CS model to previously reported group and context effects on temporal discounting. Note that we provide results from the hyperbolic model for comparison, because this model has been applied in the previous analyses of these data [49], [50]. To account for the fact that *a*- and *k*-parameters can take on values close to 0, we applied a square-root transformation to all parameters, rather than a log-transformation (see methods). Gamblers had a greater hyperbolic *k* (t_(33)_ = 2.14, p = .04, two-tailed). In the CS model, we observed a significantly increased *a*-parameter in the gamblers (t_(33)_ = 2.28, p = .023, two-tailed), whereas there was no significant group difference in *b* (t_(33)_ = 1.23, p = .23, two-tailed).

We next re-examined a within-subject comparison between a control condition and a discounting condition involving episodic future thinking. Note that in the control condition, subjects performed a standard delay discounting task (see methods). In the episodic condition, additional information regarding subject-specific future event cues was shown [49]. Note also that for this analysis we pooled across subjects from Experiments 1 and 2 of the Peters & Buchel (2010) study, yielding n = 46 in total. The hyperbolic *k* was significantly greater in the control compared to the episodic condition (t_(45)_ = 2.493, p = .016, two-tailed). In contrast, the *a*-parameter of the CS model showed no difference (t_(45)_ = 1.342, p = .186, two-tailed) whereas the b-parameter tended to be larger in the episodic condition (t_(45)_ = −1.871, p = .0679, two-tailed).

## Discussion

We applied maximum likelihood parameter estimation and model comparison techniques to examine different models of temporal discounting. We assessed the fit of a range of prominent models in a large number of datasets from healthy human subjects (n = 198). Additionally, data from pathological gamblers (n = 17) were analyzed to assess models in a clinical group with known impairments in impulse control. The analyses replicate the previous observations that dual-parameter models provide a superior fit to human discounting data than single-parameter models [10], [15], [20], [24], even when accounting for model complexity [24]. Two of the dual-parameter models (R and CS) were not examined in previous comparisons [10], [24], and one of them, the CS model [12] emerged as providing the best fit to human temporal discounting data, both for healthy participants and pathological gamblers. This result extends a previous report which examined an exponential-power model with the exponent fixed at 0.5 [14]. Results were confirmed in a number of analyses. R^2^ was greater for the CS model than any other, for both group and individual indifference point data. Also, the CS model was the “winning model” in the largest proportion of participants across different datasets. Finally, a Bayesian Model Selection analysis [56] revealed that the CS model was more likely than any other model from the set of candidate models to have generated the data of a randomly selected participant from the population.

A number of features of the CS model may prove useful in future studies. First, the model incorporates exponential discounting as a special case (*b* = 1). Degrees of deviation from “rational” or “normative” temporal discounting can thus be directly assessed. Second, our analyses confirm that the formulation of the model leads to estimates of impatience (*a*-parameter) and time-sensitivity (*b*-parameter) that are only moderately correlated. Importantly, the inter-parameter correlations observed for the CS model were considerably lower than those observed for the GM and R models. Thus, although the fit to empirical data (at least relative to single-parameter models) is improved with the GM and R models, a separate interpretation of the parameters obtained from these models is complicated by this strong dependence. Third, the CS model was found to be sensitive to both group differences in discounting between gamblers and controls, and context effects on discounting within healthy controls. Interestingly, we observed a significantly increased *a*-parameter (increased impatience) in the gamblers. Experimental manipulations that alter attention to time have been reported to affect *b* rather than *a* [12], and our data therefore suggest that diminished self-control in pathological gamblers may largely affect *a*. Despite the considerable literature on dual-parameter models (see introduction), with a few exceptions [70] the many studies examining group differences in delay discounting apply single-parameter models. One reason may be that for some well-known dual-parameter models (e.g. R, GM) group differences may be masked by the strong dependencies between parameters. In line with this idea, none of the paired comparisons between controls and gamblers for the R and GM model parameters were significant, neither when using t-tests on square-root transformed values, nor for non-parametric Wilcoxon rank-sum tests (all p>.31). In contrast, our analyses suggest that the CS model is sensitive to such group differences. With respect to contextual effects, our data suggest that episodic event cues may exert their influence via an increase in *b*, rather than a decrease in *a*. This may suggest that future event cues increase participants' sensitivity to time [12], thereby driving the discount function more in the direction of “rational” exponential discounting (i.e. the resulting *b*-parameters are closer to 1).

A recent study modelled temporal discounting as a forward search process through a representational space of future outcomes [71]. Depending on available search time and the presence of attractor basins in the energy landscape of future rewards, the model produced different discount functions. An interesting observation was that under some conditions, the discount functions revealed initial plateaus, similar to the case of b>1 in the CS model (see e.g. Ebert & Prelec 2007, Figure 1), which was observed in ≈17% of participants. The other dual-parameter models examined in this study cannot account for such behaviour.

All model comparison procedures applied in the present study are dependent upon the particular set of models examined, i.e. they test for evidence that a particular model is the “best” model *in the set of candidate models*. Conclusions are therefore always specific to the set of candidate models examined. Some discussion regarding models omitted from the comparison procedure is therefore warranted. A model by Grace [72] that includes an additive constant in the denominator of the hyperbolic equation was also examined (data not shown) but not included in the final model comparison because its' fit was lower than that of the single parameter models. Because we focussed on the single vs. dual-parameter comparison, we also did not include models with non-linear outcome scaling [7], [41]. Finally, the discounting-by-intervals model (DBI) suggests that hyperbolic discounting may occur because discounting is *subadditive* [17], [40], [73], i.e. steeper over shorter intervals, regardless of when in time these intervals occur, rather than because impatience is decreasing. In all datasets analyzed here, the delay to the larger-later reward was confounded with the interval between the two rewards (the delay to the smaller-sooner option was always 0), effectively precluding us from directly testing the DBI model [17]. However, note that when the delay to the SS reward is 0, the DBI model corresponds to hyperbolic discounting with power-scaling of time (i.e. the R model). An examination of the models by Scholten & Read [17], [73], in comparison to the models tested in this report, may therefore be an interesting issue for future modelling work on inter-temporal choice. Similarly, future studies might benefit from utilizing more sophisticated procedures for trial generation, which may improve the ability to differentiate between different underlying models [74].

Parameter estimates from mathematical discounting models have consistently been shown to be stable in individual subjects across intervals ranging from weeks to months [26], [75]–[78]. Delay discounting is therefore at least in part similar to a personality trait [5], [79]. In contrast, the decision-by-sampling model (DbS) [80] emphasizes the instability and context-dependency of preferences, including temporal discounting. DbS suggests that attributes of options (e.g. magnitudes, delays, probabilities) are subjectively weighted by comparing the attribute's actual value to a distribution drawn from memory via repeated sampling. This distribution is in turn directly affected by the immediate context in which a decision occurs [81], thereby providing a mechanism by which context may affect choice. Notably, however, these accounts (preference stability vs. context-dependency) are not mutually exclusive, as context-dependent (state) modulations likely occur relative to a stable, trait-like baseline level [3], [5], [79].

Goodness-of-fit is one important aspect of model selection, but not the only one. For example, the extent to which different models fit into a larger theoretical framework needs to be taken into account [10]. The R and CS models as well as the additive utility model [7] include a time scaling exponent and are thus compatible with Stevens psychophysical law [42]. In contrast, the GM model equation can be derived by assuming exponential discounting with subjective time *r* following the Weber-Fechner-Law, i.e. 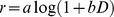 [24]. Recent empirical work has also focussed on disentangling effects of temporal discounting *per se* from effects of subjective time perception [35], [36], though these studies did not examine dual-parameter models. The analyses presented in the present report show that temporal discounting data are better accounted for by the CS model than any other model examined. In addition, our re-analyses of previously published data with the CS model provides additional insights. Loss of self-control in gamblers may affect the *a*-parameter (increased impatience) whereas reduced discounting due to episodic thinking may affect the *b*-parameter (increased temporal attention/attenuated non-linear temporal scaling). Further work is required to establish the utility of these more sophisticated discounting models in the analysis of individual differences and contextual modulations.

Taken together, our comparison of temporal discounting models confirms that dual-parameter models outperform single-parameter models, even when controlling for model complexity, complementing and extending previous model comparison studies [10], [24]. An exponential-power model (CS) [12] provided the best fit to human discounting data across a range of datasets. This result was confirmed in a BMS analysis [56], which revealed that the CS model was more likely than any other model tested to have generated the data of a randomly selected subject from the population (exceedance probability >95%). Inter-parameter correlations in the CS model were moderate compared to two other prominent dual-parameter models (GM and R), in which inter-parameter correlations were substantial. Finally, we show that the CS model is sensitive to previously reported group and context effects on temporal discounting, suggesting that it might be a useful tool in future psychiatry, psychology and neuroscience work on inter-temporal choice.
